# The intergenerational legacies of research disinvestment, aid retrenchment and transactional compacts on global women’s health

**DOI:** 10.1371/journal.pgph.0006110

**Published:** 2026-04-02

**Authors:** Roxanne C. Keynejad, Abigail M. Hatcher, Diksha Sapkota, Nataly Woollett, Simone Honikman, Alexis Palfreyman

**Affiliations:** 1 King’s Women’s Mental Health, Health Service and Population Research department, Institute of Psychiatry, Psychology & Neuroscience, King’s College London, London, United Kingdom; 2 Faculty of Health Sciences, School of Public Health, University of the Witwatersrand, Johannesburg, South Africa; 3 Gillings School of Global Public Health, University of North Carolina, Chapel Hill, United States of America; 4 Griffith Criminology Institute, Griffith University, Gold Coast, Australia; 5 Faculty of Art, Design and Architecture, University of Johannesburg, Johannesburg, South Africa; 6 Perinatal Mental Health Project, Alan J Flisher Centre for Public Mental Health, Department of Psychiatry and Mental Health, University of Cape Town, Cape Town, South Africa; 7 Centre for Impact on Violence and Health, Colombo, Sri Lanka; University of Washington Department of Global Health, UNITED STATES OF AMERICA

## Abstract

Global health funding by the United States government declined precipitously in 2025, triggering a cascade of international disinvestment in women’s health in low and middle-income countries, followed by a new era of ‘America First’ aid policy. Successive budget cuts by other high-income countries compounded the effects of these abrupt policy and geopolitical changes on women’s health, safety, and access to life saving and health assuring prevention, care and treatment, worldwide. Abandonment of active programmes and research studies disproportionately harms women by disrupting employment, reversing progress in education, eroding essential services and undermining community partnerships. The global health funding crisis weakens systems and diminishes capacity, impacting women’s physical and mental health, economic survival, and equity of opportunity across the life-course. New proposals to tie aid funding to data sharing have disproportionate impacts on women, while continued hostility to gender diversity places non-binary and transgender people at increased risk of further marginalisation. The current moment of crisis also illuminates profound limitations of the international systems which previously governed global health, indicating opportunities for transformation. In this Essay, we outline the foreseeable, intergenerational harms of defunding initiatives which promote and enhance the health and safety of women everywhere or tie their funding to agendas which would erode women’s reproductive autonomy. Amid deliberate efforts to disorientate opposition through simultaneous and sequential upheavals and concerted censorship of academic freedom, we call for concerted outcry, organised resistance, and synchronous action to safeguard women’s health and rights before more damage is done.

## Background

Events impacting global health in 2025 are by now well-described. The first news of targeted dismantling of the research system by the United States (US) government came from directly affected scientists via social networks and blogposts [1]. In relation to aid, images of USAID offices being torn apart were shared, while over 230,000 global health practitioners across 159 organisations had lost their jobs by 30^th^ May [2]. The purge of staff and programmes across global economies was expansive, with a list leaked of activities targeted for cessation, associated with diversity, equity and inclusion (DEI) efforts. Priorities for defunding were not gender neutral, with keywords ranging from ‘women’ and ‘female’, to ‘gender-based violence’ and ‘trauma’. Research cuts were cemented by further restrictions on recipients outside US borders [3]. Within months, the abrupt contraction of resources served to chill and even inspire fear of international collaboration intended to improve and save lives.

Swift decisions were made by the United Kingdom (UK) government to reduce its own aid (and global health research) budget by 40% by 2027 [4,5]. Proposals under consideration by the UK National Institute for Health and Care Research (NIHR) were frozen and in August, a new, sole focus on acute and chronic infectious diseases in Africa and South Asia was announced [6]. Similar cuts were made in parallel by Dutch, French, German and Swiss governments [7]. Reduction of the World Health Organization (WHO) annual budget by 21% (£464 million) after the US’ withdrawal [8] was only partially offset by China and other nations’ increased contributions. Overall overseas development assistance (ODA) was projected to fall by 9–17% in 2025, having fallen 9% in 2024 [9]. The world’s least developed countries are expected to lose 13–25% of their net bilateral ODA.

In September 2025, the US Department of State released its ‘America First’ Strategy [10], which was noted for neglecting maternal and child health within a narrow definition of global health [11]. While positioning global health in relation to American interests, the strategy also stated an intention to engender long-needed reforms to the aid system [12]. However, overt political motivations were apparent. With a focus on Africa, problems of reliance on aid were attributed to low and middle-income countries (LMICs) rather than flawed choices by donor countries [13]. The intention to counter the influence of China was explicitly stated.

The variety of terms secured by the 14 African countries which have thus far signed compacts under this strategy demonstrates the explicitly politicised nature of ‘America First’ global health funding, and the corresponding disregard for human health needs [14].

This overtly transactional approach to global health was followed in 2026 by a series of unilateral incursions into foreign territories, with explicit intent to acquire land, natural resources and strategic advantage [15][16]. In a new world order of imperialistic ambitions for regional hegemony [17] by the US, China and Russia, the field of global health, with talk of decolonial aspirations, is unrecognisable, one year later.

The ‘Promoting Human Flourishing in Foreign Assistance’ (PHFFA) policy recently expanded the ‘global gag rule’ (also known as the Mexico City Policy) prohibiting aid to organisations which provide abortion care [18]. PHFFA broadens its reach to all foreign assistance bar military aid, including emergency humanitarian funding, and covers organisations, institutions, and even governments. PHFFA extends beyond abortion care to any services alleged to promote “discriminatory equity ideology” or “gender ideology” [18,19].

What, then, of the human cost of these relentless, unprecedented policy shifts? It is no exaggeration to say that these events have catastrophic implications for health in LMICs. While communicable diseases have been the focus of particular concern [20,21], early responses also recognised the disproportionate impact of sudden funding withdrawals on the health of women and girls [22,23]. However, the pace and scale of disruption became hard to track. A deliberate strategy to ‘flood the zone’ with multiple, simultaneous, large-scale policy changes succeeded in inhibiting more widespread and coordinated condemnation [24]. Parallel processes sought to censor freedom of speech and academic freedoms in higher education institutions through defunding, in the guise of “defending women” [25].

As researchers and practitioners in global women’s health, a not inconsiderable number of our colleagues feel unsafe to comment publicly. Our peers and collaborators have real concerns about the ramifications of speaking out; silence cannot be interpreted as acquiescence. Since January 2025, we have grappled with the language to express our dismay and revised this Essay repeatedly, as new developments compounded initial harms. We here offer an evidence-informed perspective on the foreseeable consequences of the new world order for the health and safety of women and girls, worldwide, and actions available to each of us, across sectors and levels of influence.

### Implications for women and girls’ health and well-being

From menstruation to contraception, abortion, sexuality, fertility, pregnancy and post-partum, gynaecological conditions and menopause, women face biological and social realities carrying elevated risks of disability and ill-health, relative to men [26]. These effects have wider impacts on many children and older people, given women’s continued, disproportionate caregiving responsibilities. Dismantling global health funding without any transitional planning directly opposed international calls to intensify investment in women’s and children’s health [27]. The cessation of established activities threatens hard-won improvements across maternal, neonatal and older adult mortality from nutritional, communicable and non-communicable diseases as well as obstetric adversities [28]. For example, modelling suggests that total withdrawal of US aid funding would be associated with up to 55 million unplanned pregnancies, up to 16 million unsafe abortions and 4.5 million additional child deaths across 51 countries between 2025 and 2030 [20]. Data from 134 countries from 1990 to 2015 showed that Mexico City Policy implementation during periods of Republican governance was associated with increased maternal and child mortality and human immunodeficiency virus (HIV) incidence. Across 30 LMICs, women had less contraceptive access, family planning and HIV information and more unplanned pregnancies during periods when the global gag rule (far less restrictive than the recent PHFFA policy) was implemented [29].

Many chronic conditions, from HIV to common mental disorders (CMDs), affect women differently to men, in terms of prevalence, onset, risk factors, and social sequelae [30]. Cuts to research into conditions of ageing such as dementia and cardiovascular disease [31,32] will impact older women, who have longer life-span but lower health-span expectancies than men [33]. WHO estimates that nearly 1 in 7 people worldwide lives with a mental health condition, with women accounting for 53.1% [34]. CMDs disproportionately impact women, who experience 60.5% of depression and 62.6% of anxiety disorders [34].

In addition to established mental health inequity, the health and safety of women are intertwined with social and environmental factors which are being actively de-prioritised. Simultaneous moves to withdraw the US from Paris and United Nations (UN) climate accords and conventions [35] will have direct and indirect gendered health impacts. Women’s physical and mental health, safety and livelihoods are impacted by climate change across the life course, with particular vulnerabilities in adolescence, fertile years and older age [36,37].

As well as the WHO and UN climate treaty, the US left the UN Entity for Gender Equality and the Empowerment of Women in January 2026, stating that they “no longer serve American interests” [35]. Global initiatives to prevent gender-based violence (GBV) have been defunded as part of an anti-DEI agenda, [38] despite domestic prioritisation of the same issue. These decisions came as evidence was published that intimate partner violence (IPV) and sexual violence against children are among the top five contributors to the global burden of disease in women aged 15–49 years [39]. Thus, cuts to GBV prevention and response efforts worldwide pose risks to women and girls’ physical and mental health and safety, including femicide [40].

Harms such as violence and substance abuse amplify each other through synergistic epidemics [’syndemics’; 41]. For example, exposure to IPV is bidirectionally associated with addiction [42], depression and suicide attempts [43]. A UK birth cohort study found that physical IPV victimisation of women was associated with increased IPV perpetration by their young adult sons, after adjusting for potential confounders [44]. This finding suggests that policy making which increases the risk of GBV may serve to foment cycles of violence with intergenerational repercussions.

Mental health conditions arising in the perinatal period (from conception to one year post-partum) are also more prevalent among women facing psychosocial stressors. Examples include IPV [45,46] and forced migration [47], which interact with other adversities and often co-occur [48]. Poor perinatal mental health is in turn associated with established but not inevitable increases in psychological and developmental disorders among children [49–51], again indicating potential for intergenerational impacts.

In addition to effects on children, large populations of adolescents now face trajectories of intersecting adversity as they enter adulthood. The African region is especially affected, with more than half the population comprising children and adolescents. Young people face economic precarity and under-funded healthcare, in high fertility settings where access to contraception and abortion is restricted [52]. Although 18% of the global population lives in the African region, it accounts for over 70% of maternal deaths [53]. Despite the need for investment, health priorities such as maternal and child mortality or even population size appear to have had little bearing on the extent of global health compacts signed by 14 African countries to date [14].

The range of inter-related harms to the mental and physical health and survival of women in resource-restricted settings is highly preventable, and until 2025 had been actively mitigated by projects supported by high income countries (HICs; Box 1).

The health impacts of funding cuts will be compounded by the global move towards armed conflict, prioritising defence spending at the expense of aid and health research [54]. Contradictions between global ‘security’ and global health security are not yet being confronted. The still-recent coronavirus pandemic showed that traditional global health security strategies: national preparation, controlling borders and stockpiling were insufficient without trust, transparency and solidarity [55]. Inclusive, collaborative global health policy making is threatened by a climate of war.

The shift away from alliances for global health security and towards defence is occurring despite profound impacts of war on the health and survival of women and children [56]. Revoked funding for responsive humanitarian activities, such as camps for displaced persons, exacerbates harms further, especially for the women, children and older adults least able to fight or flee. The specific health needs of older women in such settings have been historically neglected [57]. Younger women in humanitarian settings report more mood and anxiety symptoms while older women report more age-related, physical, and neuropsychiatric symptoms, in addition to violence, economic and displacement-related difficulties [58]. Given these adversities, a shift has been advocated away from traditional camp systems for displaced persons, towards durable community-based solutions [59]. These include efforts to support people’s safe return, local integration, or resettlement, prioritising early livelihood support, legal documentation, housing solutions, and meaningful participation in decision-making [60]. However, such transitions require global health stability and funding which are now severely lacking.

**Fig 1:**
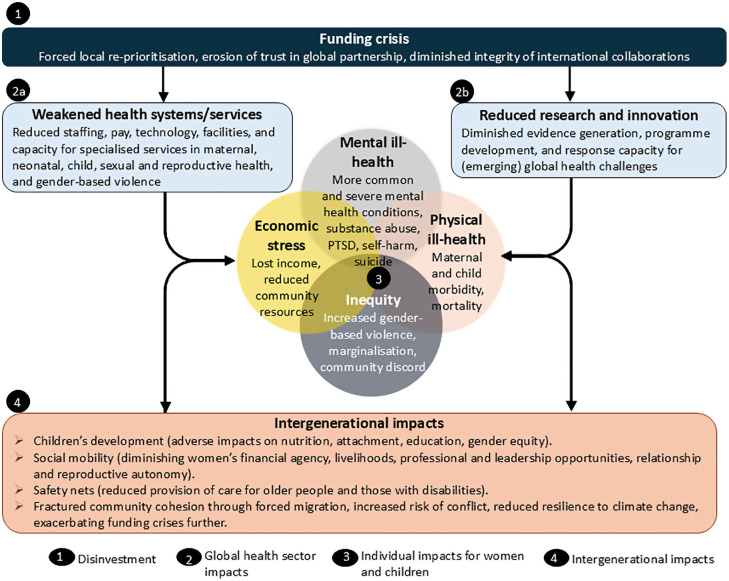
Gendered and intergenerational impacts of the abrupt withdrawal of global health funding. Alt text: A Venn diagram surrounded by boxes and arrows indicating impacts of (1) global health disinvestment on (2) the global health sector (systems/services and research/innovation). Arrows from these boxes to the Venn diagram show how (3) impacts on women and children’s mental health, physical health, economic stress and inequity lead to (4) intergenerational impacts.

Foregrounding defence at the expense of global health also disregards the community and interpersonal harms of armed conflict, through increased IPV [61] and child abuse [62], passing on trauma through families and subsequent generations. These harms are in addition to high levels of non-partner perpetrated violence [63] during and long after periods of war, with corresponding associations with mental health conditions [Fig 1; 64]. A retrospective observational study of over 35,000 survivors of violence in humanitarian settings in 11 countries found that older women reported more conflict-associated assaults by armed groups while younger women reported more sexual IPV [65]. Violent misogyny is increasingly recognised as relevant to extremism and radicalisation that lead to terrorism [66]. While the underlying causal relationships require research of the form whose funding has been cut, prioritising war and de-prioritising violence prevention will further exacerbate cycles of GBV and conflict. While mental health impacts of conflictare well-known [67], they tend to be neglected until post-war legacies of trauma become apparent, with responses often under-resourced [68].

### Box 1: Defunded research, projects, and initiatives affecting women’s and girls’ health and well-being

A selection of projects and initiatives defunded since 2025, attending to complex relationships between women and children’s physical and mental health, safety, and well-being:

*Research*

- Nationally representative household Demographic and Health Surveys (DHS) in over 90 LMICs [69], including measures of IPV, maternal and child health, and sexual and reproductive health and rights (SRHR). Interim funding covers some but not all aspects of the programme [70].
- An estimated 300 randomised controlled trials (RCTs) were abruptly terminated due to US National Institutes of Health funding cuts or pauses [71], with many focused on women’s health.
- UK NIHR global health funding streams were frozen to current and new applicants, halting the progress of a range of programmes, projects and groups addressing women’s and children’s health, and relevant social adversities. LMIC-based principal investigators lost the relatively new opportunity to apply for research fellowships, independent of a HIC-based co-applicant, a crucial step towards decolonising global health funding systems. When the scheme re-opened, new NIHR strategic global health priorities were acute and chronic infectious diseases and antimicrobial resistance in sub-Saharan Africa and South Asia, [72] with women and children’s health, mental health and non-communicable diseases notably absent.

*Projects and initiatives*

- WHO reduced its departments from 76 to 34, impacting activities addressing maternal and child health, SRHR, mental health and substance abuse, and GBV [8].
- Aid has been terminated to countries highly dependent on ODA, such as South Sudan, Somalia, and Afghanistan [73], with large-scale economic impacts on employment and livelihoods, in addition to education and healthcare programmes.
- A total of $335 million in US funding has been withdrawn from the UN Population Fund focused on reproductive health [74].
- Thousands of community health worker jobs funded by the President’s Emergency Plan for AIDS Relief have been terminated, reducing provision of HIV testing, pre- and post-exposure prophylaxis and antiretroviral treatment. In South Africa, (predominantly female) commercial sex workers have been particularly affected [75].
- Safe spaces, health and support services and violence prevention initiatives addressing GBV in Yemen, South Sudan, Central and West Africa [76], Iraq, Syria, Myanmar and Sri Lanka have had funding withdrawn [77].
- The USAID Country and Global Leadership project focused on improving maternal, newborn, and child health, family planning, and reproductive health care ceased from May 2025, halting progress on a perinatal mental health monitoring framework [78].

### Wider impacts on gender equity

In addition to direct impacts on women’s health and well-being, global health disinvestment is entrenching patriarchal, colonial dynamics of oppression, reversing decades of progress [79]. There are concerns that anti-democratic forces furthering the backlash against DEI will go further, by systematically withdrawing reproductive and civil rights of women, gender and sexual minorities [80]. Such actions are likely to impact LMICs which sign five year compacts under the ‘America First’ global health strategy, that demand 25 years’ health data sharing [81], including about abortion care [82]. Restricting women’s sexual and reproductive health and rights will impact women’s health and survival, as well as their autonomy. An analysis of WHO and UN data for 195 countries and territories assessed factors contributing to the 41% maternal mortality reduction between 2000 and 2023 [83]. The authors found that 61.2% of this fall in deaths was attributable to improved maternity care and 38.8% to reduced numbers of pregnancies.

A little over a year after Trump took office, aid cuts and the anti-DEI agenda have aligned more closely. PHFFA clearly demands policy and terminology shifts in exchange for financial support. The vagueness of terms used (“discriminatory equity ideology”, “gender ideology”) have been judged likely to bar or limit care for lesbian, gay, bisexual, transgender and queer (LGBTQ+) people in addition to women, girls, and ethnic minority groups [18]. Interference with research transparency through selective withdrawals of evidence from government websites has been used to characterise women as fragile and transgender people as threatening [84]. Targeted attacks on sexual and gender minorities will compound existing health inequities [85] and worsen health impacts of the already high prevalence of GBV victimisation of these groups [86]. Incorporation of the anti-DEI agenda into academic censorship further seeks to erase learning about gender and ethnic inequity from the next generation of researchers and professionals [25], stifling critique of structural heteropatriarchy [84,87].

Beyond effects on women as healthcare service users, cuts to global health aid and research are highly damaging to women working in roles across the field. In concrete terms, women’s psychosocial prosperity is being harmed by the elimination of global health roles and responsibilities which they overwhelmingly occupy. While women comprise 70% of global health workers, they make up only 25% of senior leaders.[88] Community health worker positions are more accessible than other positions for women with limited education and training, or who require flexibility to accommodate substantial unpaid caring responsibilities to children and elderly relatives.

Occupational segregation means that job losses have been gendered, both for health workers and scientists, with terminated positions damaging a generation of future female leaders in global health research [89] and practice. Loss of health and research roles is compounded by the contemporaneous imposition of trade tariffs, which disproportionately impact female garment workers in LMICs such as Haiti, Lesotho, Madagascar, Bangladesh, Cambodia, India and Sri Lanka [90].

Financial hardship will reverse progress towards gender equity in women’s education, leadership, and its wider, lasting benefits. In communities with especially large aid dependence, abrupt creation of large-scale job competition has the potential to foster interpersonal and intergroup conflict. Gendered job losses will exacerbate the gender pay gap and, by extension, unequal poverty among women compared to men in older age [88].

Beyond the personal impacts on research and clinical staff, the rapid withdrawal of funding has caused substantial harm to international partnership working in global health. Collaborative global health programmes and research rest on decades of relationship-building and trust developed between communities and organisations, which are the foundations for activities furthering health and gender equity. Overnight funding cessation decimates the authenticity and integrity of these partnerships through disregard for ethics and procedure. These effects will necessarily – and by design – have inequitable professional implications for majority-female practitioners and scholars on the frontline of the response.

Among the remaining global health researchers, competition for constrained funding will obstruct collaboration and worsen the pre-existing inefficiency of developing proposals with diminishing chances of success [91]. Weakening team-led scientific endeavour will harm women and children first, through reduced health discovery and advancement and second, through lost scientific education and opportunities.

### Costs to future generations

When it is fostered and valued, global collaborative research yields widespread benefits for women and children. In mental health, evidence for task-sharing in LMICs, including interventions delivered by respected female elders [92], have inspired effective new treatment models in North America [93] and the UK [94]. Research in South Africa, Tanzania, and India provided early evidence for associations between violence exposure and HIV risk, directly informing care and service design in HICs [95–99]. Trade-offs of research cuts include slowed innovation, workforce contraction, and increased health spending [100].

Beyond such direct harms, there are substantial economic and social costs of defunding efforts to prevent and mitigate GBV and mental health conditions. For illustration, domestic abuse in England and Wales alone costs an estimated £66 billion per year, primarily from physical and emotional harm to victim-survivors [101]. Mental health conditions cost the UK economy at least £118 billion per year, of which 72% comprises service users and carers’ lost work output [102]. England spends £16 billion per year to address preventable difficulties arising from adverse childhood experiences, including parental mental ill-health and substance abuse, and witnessing IPV [103]. These costs are incurred in a context without armed conflict, widespread climate events or extreme poverty and with healthcare free at the point of access. The scale of harm caused by global health disinvestment in settings without such privilege will be far greater, especially when compounded by large-scale health disinformation and misinformation [104].

While these economic quantifications are stark, we question equating the value of global health and gender equity with merely economic gains. The UN special rapporteur on extreme poverty contends that undue focus on gross domestic product exacerbates inequity and degrades environments [105]. De Schutter instead advocates a ‘human rights economy’ which centres population and planetary well-being within decision-making. While global aid cuts have been attributed to electorates’ domestic concerns, around 60% of Americans advocate for investment in activities which strengthen economic development and democracy abroad [106].

### Collective action

Many commentaries on recent events conclude by advocating a reckoning with a neocolonial aid system, a stance that does not always confront the scale of loss to women’s and children’s lives [107]. Yet those of us leading organisations receiving aid do identify inefficiencies and organisational structures which absorbed vast resources without transforming lives on the ground. Insufficient aid reached intended beneficiaries, with large sums profiting pharmaceutical companies [108,109] or expended on administrative overheads and executive salaries. Disbursement of aid via overseas-led non-governmental organisations (NGOs) missed opportunities to strengthen the capacity of local health systems and institutions and left countries vulnerable when funding was withdrawn [110]. Global health research and programming suffer from bureaucracy and misaligned incentives which distract from their purpose. Power imbalances persist throughout the system [111], with resources flowing to places that are politically expedient or well-connected rather than in greatest need. Our colleagues, whose communities and livelihoods are directly affected, are grappling with two truths. The global health funding crisis is creating real human devastation, but redesigning the system, which has failed to deliver sustainable change, is also overdue. Table 1 summarises potential responses to the systemic problems facing different global health sectors and those with power and/or responsibility to enact them.

**Table 1:** Potential responses to systemic problems facing global health sectors and key actors.

Sector	Systemic problems	Potential solutions	Actors
Cross-sectoral	Neocolonial power dynamics and priority setting [13–15]	Reflect and reconceptualise power dynamics and decision-making processes [79]. Shift power from HIC centres to regional leadership [112].	All actors holding financial, epistemic and other forms of power in global health
	Marginalisation of minoritised perspectives	Vocal and practical support for advocacy and lived experience movements and integration into planning and implementation [80,113]	
	Large-scale loss of jobs and institutional expertise [2]	Alternative funding sources which recognise the value of training and jobs disproportionately occupied by women.	Government, philanthropic and other funders
		Matchmake projects to prospective funders.[114]	Professionals with contacts in different fields
	Political decision-making in HICs out of step with public preferences [106]	More vocal publicity for the small costs and large benefits of global health and aid spending. Document and quantify costs of disinvestment in global health and research [100]	Aid and research organisations and actors
	Uniformly numerical conceptualisation of global health and aid	Invest in and research novel approaches such as the Roadmap for Eradicating Poverty Beyond Growth [105]	Funders and researchers
	Intergenerational harms of investing in defence at the expense of preventative interventions [56,63,67]	Prioritise prevention, family planning and psychosocial support rather than waiting for morbidities to arise post-conflict [68]. Centre prevention and responses to GBV within global health policy and planning [66].	Funded charitable, government and research organisations. Policy makers and planners.
	The rise of misinformation [115]	Concerted efforts to combat misinformation which erodes trust in science and contributes to hesitancy towards evidence-based healthcare.	All global health stakeholders with any local, regional, national or international platform.
	Pervasive corruption [116]	Nuanced, multi-sectoral consensus and commitment to transparency, oversight and regulation at local, national and international levels.	
Civil society	Profound harms of abrupt cuts [20,117]	Vocal opposition by those with the power, privilege and platform to speak out [113].	All who feel safe and able to speak out
		Legal challenges [118].	Representative bodies
		Cancellation [119] or restructuring [120] of national debts, solidarity [121] and global transaction taxes [112] to facilitate national responsibility for healthcare funding.	International organisations with national governments
Aid	Corporate profit	Accords to facilitate cooperation to share benefits of research equitably [109].	International representative organisations
		Progressive approaches to global sharing of health innovation to avoid favouring corporate interests [108].	
	Insufficient aid reaches beneficiaries	Agreed thresholds for the proportion of donor agency spending on direct aid funding.	Representative bodies
	Substitution of country functions without state engagement or oversight [121]	Minimal substitution in humanitarian and very low resource contexts and for excluded groups, with clear trajectories to transition to local ownership and delivery [121]	Aid organisations in partnership with local governments
	Disproportionate investment in disease-specific vertical programmes	Emphasis on health system strengthening and integration of women’s physical and mental health across primary care [112]	
	Lack of coordination between disjointed initiatives	Locally-led, multisectoral partnerships bringing together internally-prioritised external expertise as needed.	Charitable and aid sectors in partnership with national governments
	Wasted funds, misaligned incentives	Transition to leaner models of charitable governance with reduced executive and HIC personnel costs and more direct allocation of funds to in-country leadership and delivery. Investment in locally prioritised ‘global public goods’: in-country resources such as research, guidelines, data monitoring and procurement, and international goods such as infectious disease control and surveillance [121].	
Research	Inefficiency of increased competition for global health funding [91]	Long-term funding which strengthens local capacity and leadership and creates opportunities to researchers from under-represented groups.	Funders
	Disproportionate investment in a minority of settings	Collaborative processes at a national level to identify needs and locations requiring investment.	Researchers
	Decimation of long-term partnerships between institutions and organisations	Active efforts to remotely mentor and support early career staff during phases of funding retrenchment.	Staff with secure positions
		Maintenance of partnerships remotely until funding can be secured.	
		Ethical approaches to unavoidable project closures [107].	

If transformation of arrangements that perpetuate inequity is our goal, the abandonment of global health by the US, some European nations and multilaterals is forcing a painful, uncoordinated reset. While we should not accept the scores of preventable mortality and morbidity that will arise from this episode of destruction [20,117], we can recognise that this crisis might provide an opportunity to rethink, rebuild, and reclaim [122]. Rather than simply “documenting collapse”, research expertise can harness systems thinking to examine how complex systems are responding to unfolding external shocks [123]. Ultimately, global health requires more accountable solutions grounded in long-term, multisectoral partnerships [124,125]. Radical changes to global financing such as debt cancellation [119] or restructuring [120] and solidarity [121] or global transaction taxes [112] must now be contemplated. Worldwide corruption across global health must be confronted, definitively [116]. The central importance of women’s health, in addition to that of children and adolescents, must be prioritised by LMIC decision-makers [126], despite pivots by HICs towards infectious diseases with potential to impact them directly.

The success of fields allied to women’s health is grounded in advocacy movements [113], including women’s and feminist funds [127], the skills of which were, arguably, lost when the gender and development field became institutionalised [128]. Although speaking up and challenging authority are uncomfortable for many, those with the capacity and platform to publicly critique disinvestment in global health and DEI-furthering activities have a responsibility to do so [129]. We can ensure that the experiences and perspectives of colleagues, service users, community members, and survivors who may be at risk from speaking out are heard and considered in policy debates. We may need to gain skills and learn new languages of allyship to convey the value of projects and activities to new audiences. While much damage has been done, opposition may yet take effect [118]. The movement to #DefendResearch is one example of resistance to censorship and protection of academic freedom garnering widespread support [130,131].

More donors, philanthropists [132–134], governments and agencies must harness opportunities to fund programmes and projects fostered over decades, before all expertise and institutional memory are lost. Actions of unemployed global health staff to matchmake valuable projects with prospective funders demonstrate the persistence of the prosocial [114]. However, reimagining ineffective power dynamics requires funders to develop humility and curiosity to learn from frontline implementers. There could not be a better time to invest in locally-led and prioritised global health initiatives, in meaningful partnership. Now is an unprecedented moment, in which a cascade of adversities may yet be averted, with benefits for generations to come.

## References

[1] Saxbe D. An Attack on “Woke” Science Or an Attack on All Science? The Trump administration's attempt to cancel science. 2025 [12/06/2025]. Available from: https://darbysaxbe.substack.com/p/an-attack-on-woke-science-or-an-attack

[2] USAID Stop-Work. USAID Stop-Work. 2025 [29/07/2025]. Available from: https://www.usaidstopwork.com/

[3] NIH. Updated NIH Policy on Foreign Subawards. 2025 [27/06/2025]. Available from: https://grants.nih.gov/grants/guide/notice-files/NOT-OD-25-104.html#:~:text=NIH%20plans%20to%20expand%20this,implementing%20the%20new%20award%20structure.&text=Effective%20with%20the%20date%20of,reflect%20the%20new%20award%20structure

[4] Hanlon C, Pickersgill M, Amanfo S, Campbell H, Chiumento A, Collin J, et al. No health without aid and development. Lancet Glob Health. 2025;13(5):e803. doi: 10.1016/S2214-109X(25)00111-1 40120595

[5] UK Parliament (2026). Official Development Assistance (ODA) programme allocations 2026/27 – 2028/29. Accessed on 27/03/2026 from: https://questions-statements.parliament.uk/written-statements/detail/2026-03-19/hcws1425

[6] NIHR. Global Health Research. 2025 [20/10/2025]. Available from: https://www.nihr.ac.uk/research-funding/global-health

[7] Huckstep S, Granito L, Casadevall Bellés S, Crawfurd L. Charting the Fallout of Aid Cuts: Which Countries Will be Hit Hardest, as Multiple Donors Cut Budgets? 2025 [23/06/2025]. Available from: https://www.cgdev.org/blog/charting-fallout-aid-cuts

[8] Taylor L. WHO to cut budget by a fifth following Trump withdrawal. BMJ. 2025;389:r651. doi: 10.1136/bmj.r651 40169201

[9] OECD. Cuts in official development assistance: OECD projections for 2025 and the near term Paris, France. OECD Publishing; 2025 [26/10/2025]. Available from: https://www.oecd.org/en/publications/cuts-in-official-development-assistance_8c530629-en/full-report.html

[10] U.S. Department of State. America First Global Health Strategy. 2025 [08/02/2026]. Available from: https://www.state.gov/america-first-global-health-strategy

[11] Modernizing Foreign Assistance Network. MFAN statement on the administration’s new global health strategy. 2025 [08/02/2026]. Available from: https://www.modernizeaid.net/press-room/mfan-statement-on-the-administrations-new-global-health-strategy

[12] Morrison JS, Gostin LO. Promise and gaps in America First strategy for global health. Br Med J Publish Group. 2025. 10.1136/bmj.r2088 41057152

[13] Okereke E. America first in global health: How Africa should respond. Think Global Health; 2025 [08/02/2026]. Available from: https://www.thinkglobalhealth.org/article/america-first-in-global-health-how-africa-should-respond

[14] Aghogho N. Leverage and constraint: African agency under the America First global health strategy. Global Policy; 2026 [08/02/2026]. Available from: https://www.globalpolicyjournal.com/blog/05/02/2026/leverage-and-constraint-african-agency-under-america-first-global-health-strategy

[15] Hardy AT. Venezuela: The beginning of the hunting season. 2026 [08/02/2026]. Available from: https://www.globalpolicyjournal.com/blog/08/01/2026/venezuela-beginning-hunting-season

[16] Immerwahr (2026). What’s behind Trump’s new world disorder? New Yorker. Accessed on 27/03/2026 from: https://www.newyorker.com/magazine/2026/03/23/whats-behind-trumps-new-world-disorder

[17] Mearsheimer JJ. Anarchy and the Struggle for Power. The Realism Reader. Routledge; 2014. p. 179–87.

[18] Medecins Sans Frontieres. MSF condemns sweeping expansion of the Global Gag Rule. 2026 [15/02/2026]. Available from: https://www.doctorswithoutborders.org/latest/msf-condemns-sweeping-expansion-global-gag-rule

[19] Burkybile F. Why the expanded global gag rule is a deadly triple tripwire for recipients of US foreign aid. BMJ. 2026;392:s528. doi: 10.1136/bmj.s528 41881457

[20] Stover J, Sonneveldt E, Tam Y, Horton KC, Phillips AN, Smith J, et al. Effects of reductions in US foreign assistance on HIV, tuberculosis, family planning, and maternal and child health: a modelling study. Lancet Glob Health. 2025;13(10):e1669–80. doi: 10.1016/S2214-109X(25)00281-5 40975076 PMC12447089

[21] da Silva AF, Anderle RVR, Sibils GB, de Oliveira Ferreira de Sales L, Pena D, Monti C, et al. Impact of two decades of humanitarian and development assistance and the projected mortality consequences of current defunding to 2030: retrospective evaluation and forecasting analysis. Lancet Glob Health. 2026;:S2214-109X(26)00008-2. doi: 10.1016/S2214-109X(26)00008-2 41643687

[22] Clark J. The war on equality. BMJ. 2025;388:r508. doi: 10.1136/bmj.r508

[23] Martinez-Alvarez M, Amouzou A, Barros AJD, Faye C, Behanzin P, Borghi J, et al. Broken promises: the USA foreign aid freeze threatens women’s, children’s, and adolescents’ health. Lancet. 2025;405(10488):1448–50. doi: 10.1016/S0140-6736(25)00558-6 40222383

[24] Montgomery B. How Musk and Trump are flooding the zone. The Guardian; 2025 [26/10/2025]. Available from: https://www.theguardian.com/technology/2025/feb/11/musk-trump-doge-ice-google-seo-spotify

[25] Reid A, Friedman J, Sachs JA, Benitez L. America’s censored campuses 2025: Expanding the Web of Control. PEN America; 2026 [15/02/2026]. Available from: https://cdn.pen.org/wp-content/uploads/2026/02/10104944/Americas-Censored-Campuses-2025_02-10-26.pdf?_gl=1*qa49h*_gcl_au*MTc4MTI3MjE4My4xNzcxMTQ3Njcz*_ga*MTQzMzM3NjM1Ni4xNzUxMDM5MjAz*_ga_0RQGYGDH22*czE3NzExNDc2NzQkbzIkZzAkdDE3NzExNDc2ODgkajQ2JGwwJGgxNDMwOTkwNTY3

[26] Howard LM, Wilson CA, Reilly TJ, Moss KM, Mishra GD, Coupland-Smith E, et al. Women’s reproductive mental health: currently available evidence and future directions for research, clinical practice and health policy. World Psychiatry. 2025;24(2):196–215. doi: 10.1002/wps.21305 40371748 PMC12079463

[27] Devex Partnerships. Reigniting momentum for maternal, newborn, and child health. 2025 [30/06/2025]. Available from: https://www.devex.com/news/sponsored/reigniting-momentum-for-maternal-newborn-and-child-health-110290

[28] WHO. Aid cuts threaten fragile progress in ending maternal deaths, UN agencies warn. 2025 [27/06/2025]. Available from: https://www.who.int/news/item/07-04-2025-aid-cuts-threaten-fragile-progress-in-ending-maternal-deaths-un-agencies-warn

[29] Kavakli KC, Rotondi V. US foreign aid restrictions and maternal and children’s health: Evidence from the “Mexico City Policy”. Proc Natl Acad Sci U S A. 2022;119(19):e2123177119. doi: 10.1073/pnas.2123177119 35500117 PMC9171610

[30] Barr E, Marshall LJ, Collins LF, Godfrey C, St Vil N, Stockman JK, et al. Centring the health of women across the HIV research continuum. Lancet HIV. 2024;11(3):e186–94. doi: 10.1016/S2352-3018(24)00004-3 38417977 PMC11301651

[31] The Lancet Healthy Longevity. The cost of US funding cuts. Lancet Healthy Longev. 2025;6(3):100703. doi: 10.1016/j.lanhl.2025.100703 40058386

[32] Tanne JH. Trump U turns on scrapping of US’s largest women’s health study. Br Med J. 2025. 10.1136/bmj.r831 40280596

[33] Garmany A, Terzic A. Global Healthspan-Lifespan Gaps Among 183 World Health Organization Member States. JAMA Netw Open. 2024;7(12):e2450241. doi: 10.1001/jamanetworkopen.2024.50241 39661386 PMC11635540

[34] WHO. World mental health today: latest data. 2025 [19/09/2025]. Available from: https://iris.who.int/bitstream/handle/10665/382343/9789240113817-eng.pdf

[35] Taylor L. Trump withdraws US from 66 global organisations and UN climate treaty. 2026. 10.1136/bmj.s65 41526045

[36] Rothschild J, Haase E. The mental health of women and climate change: Direct neuropsychiatric impacts and associated psychological concerns. Int J Gynaecol Obstet. 2023;160(2):405–13. doi: 10.1002/ijgo.14479 36165632

[37] Anjum G, Aziz M. Climate change and gendered vulnerability: A systematic review of women’s health. Womens Health (Lond). 2025;21:17455057251323645. doi: 10.1177/17455057251323645 40071991 PMC11905046

[38] Care International. Joint statement: The UK must not abandon women and girls in crisis. 2025 [30/06/2025]. Available from: https://www.careinternational.org.uk/press-office/press-releases/joint-statement-the-uk-must-not-abandon-women-and-girls-in-crisis/

[39] Flor LS, Spencer CN, Cagney J, Gil GF, Aalruz H, Abd ElHafeez S, et al. Disease burden attributable to intimate partner violence against females and sexual violence against children in 204 countries and territories, 1990–2023: a systematic analysis for the Global Burden of Disease Study 2023. Lancet. 2026;407(10523):31–52. 41386261 10.1016/S0140-6736(25)02503-6 PMC12775558

[40] Jewkes R, Abrahams N. Global burden of disease from intimate partner violence against women and sexual violence against children: a call to action. Lancet. 2026;407(10523):2–3. doi: 10.1016/S0140-6736(25)02598-X 41483900

[41] Chavez JV, Wang P, Larson ME, Vazquez V, De La Rosa M, Behar-Zusman V. Methodologies used in studies examining substance abuse, violence and HIV/AIDS (SAVA) constructs using a syndemic framework: a scoping review. AIDS Care. 2023;35(11):1708–15. doi: 10.1080/09540121.2023.2176426 36942772 PMC10511665

[42] Devries KM, Child JC, Bacchus LJ, Mak J, Falder G, Graham K, et al. Intimate partner violence victimization and alcohol consumption in women: a systematic review and meta-analysis. Addiction. 2014;109(3):379–91. doi: 10.1111/add.12393 24329907

[43] Devries KM, Mak JY, Bacchus LJ, Child JC, Falder G, Petzold M, et al. Intimate partner violence and incident depressive symptoms and suicide attempts: a systematic review of longitudinal studies. PLoS Med. 2013;10(5):e1001439. doi: 10.1371/journal.pmed.1001439 23671407 PMC3646718

[44] Herbert A, Barter C, Szilassy E, Heron J, Fraser A, Barnes M, et al. The impact of parental intimate partner violence and abuse (IPVA) on young adult relationships: a UK general population cohort study. Lancet Reg Health Eur. 2025;53:101278. doi: 10.1016/j.lanepe.2025.101278 40636059 PMC12237738

[45] Keynejad RC, Wilson CA, Howard LM. Domestic violence and perinatal mental health. Key topics in perinatal mental health. Springer; 2022. p. 421–33.

[46] Ngah Obama FX. Gender inequality, women’s human capital and female suicide in selected MENA countries. SSM Popul Health. 2025;30:101819. doi: 10.1016/j.ssmph.2025.101819 40510026 PMC12159230

[47] Stevenson K, Fellmeth G, Edwards S, Calvert C, Bennett P, Campbell OMR, et al. The global burden of perinatal common mental health disorders and substance use among migrant women: a systematic review and meta-analysis. Lancet Public Health. 2023;8(3):e203–16. doi: 10.1016/S2468-2667(22)00342-5 36841561

[48] Waterman EA, McLain M, Zulfiqar H, Ahmar Qadeer T, Ciavoi S-M. The Link Between Intimate Partner Violence and Food Insecurity: A Review of Quantitative and Qualitative Studies. Trauma Violence Abuse. 2024;25(2):1511–30. doi: 10.1177/15248380231186152 37485673

[49] Stein A, Pearson RM, Goodman SH, Rapa E, Rahman A, McCallum M, et al. Effects of perinatal mental disorders on the fetus and child. Lancet. 2014;384(9956):1800–19. doi: 10.1016/S0140-6736(14)61277-0 25455250

[50] Verkuijl NE, Richter L, Norris SA, Stein A, Avan B, Ramchandani PG. Postnatal depressive symptoms and child psychological development at 10 years: a prospective study of longitudinal data from the South African Birth to Twenty cohort. Lancet Psychiatry. 2014;1(6):454–60. doi: 10.1016/S2215-0366(14)70361-X 26361200

[51] Orri M, Besharati S, Ahun MN, Richter LM. Analysis of Maternal Postnatal Depression, Socioeconomic Factors, and Offspring Internalizing Symptoms in a Longitudinal Cohort in South Africa. JAMA Netw Open. 2021;4(8):e2121667. doi: 10.1001/jamanetworkopen.2021.21667 34410394 PMC8377574

[52] Amouzou A, Barros AJD, Requejo J, Faye C, Akseer N, Bendavid E, et al. The 2025 report of the Lancet Countdown to 2030 for women’s, children’s, and adolescents’ health: tracking progress on health and nutrition. Lancet. 2025;405(10488):1505–54. doi: 10.1016/S0140-6736(25)00151-5 40222381

[53] Martin A, Ridout A, Afolabi BB, Shennan A. The Escalating Inequalities in Global Maternal Mortality-Time to Act: A Commentary. BJOG. 2025;132(13):1928–30. doi: 10.1111/1471-0528.18246 40485436 PMC12592768

[54] Buse K, McKee M. Defence or aid, or defence and aid? Both are necessary for national security. BMJ. 2025;389:r1084. doi: 10.1136/bmj.r1084 40409785

[55] Osborne A. From preparedness to solidarity reimagining global health security post-COVID-19. BMJ Glob Health. 2025;10(9). 10.1136/bmjgh-2025-021178 PMC12414183 40912734

[56] Bendavid E, Boerma T, Akseer N, Langer A, Malembaka EB, Okiro EA, et al. The effects of armed conflict on the health of women and children. Lancet. 2021;397(10273):522–32. doi: 10.1016/S0140-6736(21)00131-8 33503456 PMC7612212

[57] van Boetzelaer E, Rathod L, Keating P, Pellecchia U, Sharma S, Nickerson J, et al. Health needs of older people and age-inclusive health care in humanitarian emergencies in low-income and middle-income countries: a systematic review. Lancet Healthy Longev. 2025;6(1):100663. doi: 10.1016/j.lanhl.2024.100663 39746372

[58] van Boetzelaer E, Sleit R, Rodriguez E, Barathi GK, Escobio F, Browne JL, et al. Differences in mental health between younger and older adults in complex humanitarian settings in low-income and middle-income countries: retrospective analysis from Médecins Sans Frontières-supported mental health services, 2019–2024. BMJ Glob Health. 2025;10(9). 10.1136/bmjgh-2025-019822 PMC12481304 41005812

[59] OECD. Refugees and internally displaced persons in development planning: No-one left behind? OECD Publishing; 2023 [16/02/2026]. Available from: https://www.oecd.org/content/dam/oecd/en/publications/reports/2023/10/refugees-and-internally-displaced-persons-in-development-planning_26eb8a6f/08c021b0-en.pdf

[60] UNHCR. Focus area strategic plan for protection and solutions for internall displaced people 2024-2030. 2024 [16/02/2026]. Available from: https://www.unhcr.org/sites/default/files/2024-08/focus-area-strategic-plan-idps-2024-2030.pdf

[61] Le K, Nguyen M. War and intimate partner violence in Africa. Sage Open. 2022;12(2):21582440221096427.

[62] Kadir A, Shenoda S, Goldhagen J. Effects of armed conflict on child health and development: A systematic review. PLoS One. 2019;14(1):e0210071. doi: 10.1371/journal.pone.0210071 30650095 PMC6334973

[63] Murphy M, Smith ER, Chandarana S, Ellsberg M. Experience of intimate partner violence and non-partner sexual violence in conflict-affected settings: a systematic review and meta-analysis. Trauma Violence Abuse. 2024. doi: 15248380241305355 10.1177/15248380241305355 PMC12569134 39717938

[64] Devakumar D, Palfreyman A, Uthayakumar-Cumarasamy A, Ullah N, Ranasinghe C, Minckas N, et al. Mental health of women and children experiencing family violence in conflict settings: a mixed methods systematic review. Confl Health. 2021;15(1):74. doi: 10.1186/s13031-021-00410-4 34654456 PMC8518246

[65] Van Boetzelaer E, Biru T, Idris A, Keating P, Staunton M, Escobio F, et al. Differences in sexual violence against younger and older adults in complex humanitarian settings: a retrospective analysis from Médecins Sans Frontières in 2019–24. Lancet Glob Health. 2025. 10.1016/S2214-109X(25)00318-3 41110454

[66] Organization for Security and Co-operation in Europe. The Linkages between violent misogyny and violent extremism and radicalization that lead to terrorism. 2022 [26/10/2025]. Available from: https://www.osce.org/files/f/documents/d/c/525297.pdf

[67] Charlson F, van Ommeren M, Flaxman A, Cornett J, Whiteford H, Saxena S. New WHO prevalence estimates of mental disorders in conflict settings: a systematic review and meta-analysis. Lancet. 2019;394(10194):240–8. doi: 10.1016/S0140-6736(19)30934-1 31200992 PMC6657025

[68] Kamali M, Munyuzangabo M, Siddiqui FJ, Gaffey MF, Meteke S, Als D, et al. Delivering mental health and psychosocial support interventions to women and children in conflict settings: a systematic review. BMJ Glob Health. 2020;5(3):e002014. doi: 10.1136/bmjgh-2019-002014 32201624 PMC7073823

[69] Khaki JJ, Molenaar J, Karki S, Olal E, Straneo M, Mosuse MA, et al. When health data go dark: the importance of the DHS Program and imagining its future. BMC Med. 2025;23(1):241. doi: 10.1186/s12916-025-04062-6 40275318 PMC12023666

[70] The DHS Program. News about the DHS Program. 2025 [20/08/2025]. Available from: https://www.dhsprogram.com/Who-we-are/News-about-the-DHS-Program.cfm

[71] Hwang I, Huang J, Anthes E, Migliozzi B, Mueller B. The disappearing funds for global health. 2025 [cited 27/06/2025]. Available from: https://www.nytimes.com/interactive/2025/06/04/health/trump-cuts-nih-grants-research.html

[72] NIHR. NIHR announces global health research calls for 2026. 2026 [07/02/2026]. Available from: https://www.nihr.ac.uk/news/nihr-announces-global-health-research-calls-2026

[73] Development CfG. Which Countries Are Most Exposed to US Aid Cuts; And What Other Providers Can Do. 2025 [13/06/2025]. Available from: https://www.cgdev.org/blog/which-countries-are-most-exposed-us-aid-cuts-and-what-other-providers-can-do

[74] UNFPA. United States Government cuts future funding for UNFPA. 2025 [27/06/2025]. Available from: https://www.unfpa.org/united-states-government-cuts-future-funding-unfpa

[75] Human L. HIV activists urge Parliament to respond to PEPFAR cuts. 2025 [27/06/2025]. Available from: https://groundup.org.za/article/activists-demand-governments-plans-to-deal-with-health-funding-cut-crisis-be-shared-publicly/

[76] Emaojo EF. Global gender-based violence response in collapse: Funding cuts push millions beyond reach of help. 2025 [24/06/2025]. Available from: https://www.developmentaid.org/news-stream/post/195746/gbv-funding-crisis

[77] Howard S. Nearly half of women’s aid organisations working in crisis areas are at risk of closure, UN survey finds. BMJ. 2025;389:r1020. doi: 10.1136/bmj.r1020 40379446

[78] Layton E, Roddy Mitchell A, Kennedy E, Moran AC, Palestra F, Chowdhary N, et al. Maternal mental health matters: Indicators for perinatal mental health-A scoping review. PLoS One. 2025;20(1):e0317998. doi: 10.1371/journal.pone.0317998 39869575 PMC11771939

[79] Nassiri-Ansari T, Gall A, Oti SO, Abdulrahim S, Rhule ELM. Decolonising women’s health innovation. BMJ. 2025;391:e085683. doi: 10.1136/bmj-2025-085683 41073083 PMC12509993

[80] Harper C, Khan A, Browne E, Michalko J, Tant E. There’s not enough money – so why spend it on gender equality and justice?. ODI Global; 2025 [26/10/2025]. Available from: https://odi.org/en/insights/theres-not-enough-money-so-why-spend-it-on-gender-equality-and-justice/

[81] Cullinan K. Kenya’s high court suspends US health deal as civil society urges African leaders to ensure ‘fair terms’. Health Policy Watch; 2025 [08/02/2026]. Available from: https://healthpolicy-watch.news/kenyas-high-court-suspends-us-health-deal-as-civil-society-urges-african-leaders-to-ensure-fair-terms/

[82] Washington J. Trump wants to make African countries share abortion data to get AIDS funding. The Intercept; 2025 [08/02/2026]. Available from: https://theintercept.com/2025/12/01/pepfar-hiv-abortion-health-data-trump/?utm_medium=email&utm_source=The%20Intercept%20Newsletter

[83] Ahmed S, Ali M, Shah I, Tsui A. Effect of maternity care improvement, fertility decline, and contraceptive use on global maternal mortality reduction between 2000 and 2023: results from a decomposition analysis. Lancet Glob Health. 2026;14(1):e33–48. doi: 10.1016/S2214-109X(25)00409-7 41192459 PMC12689492

[84] Homan PA, Short SE. Rewriting women’s health: a content analysis of the Trump administration’s revisions to womenshealth.gov. Lancet Reg Health Am. 2025;51:101288. doi: 10.1016/j.lana.2025.101288 41246550 PMC12613099

[85] Zeeman L, Sherriff N, Browne K, McGlynn N, Mirandola M, Gios L, et al. A review of lesbian, gay, bisexual, trans and intersex (LGBTI) health and healthcare inequalities. Eur J Public Health. 2019;29(5):974–80. doi: 10.1093/eurpub/cky226 30380045 PMC6761838

[86] McLellan C, Yeh PT, Kennedy CE, Barré-Quick M, Rich AJ, Poteat T, et al. Global Burden of Violence Against Transgender and Gender-Diverse Adults: A Systematic Review and Meta-Analysis. JAMA Netw Open. 2026;9(1):e2552953. doi: 10.1001/jamanetworkopen.2025.52953 41563760

[87] Scott JW. Gender studies courses are shutting down across the US. The Epstein files reveal why. The Guardian; 2026 [16/02/2026]. Available from: https://www.theguardian.com/commentisfree/2026/feb/13/gender-studies-trump-epstein

[88] WHO. Delivered by women, led by men: A gender and equity analysis of the global health and social workforce. Geneva, Switzerland; 2019 [26/10/2025]. Available from: https://cdn.who.int/media/docs/default-source/health-workforce/delivered-by-women-led-by-men.pdf?sfvrsn=94be9959_2

[89] Patel V. A death foretold. Lancet. 2025;406(10498):22–3. doi: 10.1016/s0140-6736(25)01231-0 40513589

[90] Agarwal P. Beyond trade wars: women garment workers bear the brunt of US tariff recalibration. ODI Global; 2025 [20/08/2025]. Available from: https://odi.org/en/insights/beyond-trade-wars-women-garment-workers-bear-the-brunt-of-us-tariff-recalibration/

[91] Naddaf M. Is academic research becoming too competitive? Nature examines the data. Nature. 2025;646(8087):1036–7. doi: 10.1038/d41586-025-03119-z 41107586

[92] Chibanda D, Bowers T, Verhey R, Rusakaniko S, Abas M, Weiss HA, et al. The Friendship Bench programme: a cluster randomised controlled trial of a brief psychological intervention for common mental disorders delivered by lay health workers in Zimbabwe. Int J Ment Health Syst. 2015;9:21. doi: 10.1186/s13033-015-0013-y 27408619 PMC4940904

[93] Singla DR, Silver RK, Vigod SN, Schoueri-Mychasiw N, Kim JJ, La Porte LM, et al. Task-sharing and telemedicine delivery of psychotherapy to treat perinatal depression: a pragmatic, noninferiority randomized trial. Nat Med. 2025;31(4):1214–24. doi: 10.1038/s41591-024-03482-w 40033113 PMC12003186

[94] Husain N, Lunat F, Lovell K, Miah J, Chew-Graham CA, Bee P, et al. Efficacy of a culturally adapted, cognitive behavioural therapy-based intervention for postnatal depression in British south Asian women (ROSHNI-2): a multicentre, randomised controlled trial. Lancet. 2024;404(10461):1430–43. doi: 10.1016/S0140-6736(24)01612-X 39396350

[95] Dunkle KL, Jewkes RK, Brown HC, Gray GE, McIntryre JA, Harlow SD. Gender-based violence, relationship power, and risk of HIV infection in women attending antenatal clinics in South Africa. Lancet. 2004;363(9419):1415–21. doi: 10.1016/S0140-6736(04)16098-4 15121402

[96] Silverman JG, Decker MR, Saggurti N, Balaiah D, Raj A. Intimate partner violence and HIV infection among married Indian women. JAMA. 2008;300(6):703–10. doi: 10.1001/jama.300.6.703 18698068

[97] Maman S, Mbwambo JK, Hogan NM, Kilonzo GP, Campbell JC, Weiss E, et al. HIV-positive women report more lifetime partner violence: findings from a voluntary counseling and testing clinic in Dar es Salaam, Tanzania. Am J Public Health. 2002;92(8):1331–7. doi: 10.2105/ajph.92.8.1331 12144993 PMC1447239

[98] Sareen J, Pagura J, Grant B. Is intimate partner violence associated with HIV infection among women in the United States? Gen Hosp Psychiatry. 2009;31(3):274–8. 19410107 10.1016/j.genhosppsych.2009.02.004

[99] The White House Office of the Press Secretary. Presidential Memorandum -- Establishing a Working Group on the Intersection of HIV/AIDS, Violence Against Women and Girls, and Gender-related Health Disparities. 2012 [27/06/2025]. Available from: https://obamawhitehouse.archives.gov/the-press-office/2012/03/30/presidential-memorandum-establishing-working-group-intersection-hivaids-?utm_source=chatgpt.com

[100] Jalali MS, Hasgul Z, editors. Potential trade-offs of proposed cuts to the US National Institutes of Health. JAMA Health Forum. American Medical Association; 2025. 10.1001/jamahealthforum.2025.2228 40711777

[101] Oliver R, Alexander B, Roe S, Wlasny M. The economic and social costs of domestic abuse. Home Office; 2019 [13/06/2025]. Available from: https://assets.publishing.service.gov.uk/media/5f637b8f8fa8f5106d15642a/horr107.pdf

[102] McDaid D, Park AL. The economic case for investing in the prevention of mental health conditions in the UK. Care Policy and Evaluation Centre, Department of Health Policy, London School of Economics and Political Science Mental Health Foundation; 2022.

[103] Royal Foundation Centre for Early Childhood. Big Change Starts Small. 2021 [24/07/2025]. Available from: https://assets.ctfassets.net/qwnplnakca8g/2iLCWZESD2RLu24m443HUf/1c802df74c44ac6bc94d4338ff7ac53d/RFCEC_BCCS_Report_and_Appendices.pdf

[104] Gedeon J. RFK Jr’s ‘Maha’ report found to contain citations to nonexistent studies. The Guardian. 2025.

[105] De Schutter O. A Roadmap for Eradicating Poverty Beyond Growth. 2025 [23/06/2025]. Available from: https://www.srpoverty.org/2025/01/13/a-roadmap-for-eradicating-poverty-beyond-growth/

[106] Pew Research Center. Majorities of Americans Support Several – But Not All – Types of Foreign Aid. 2025 [23/06/2025]. Available from: https://www.pewresearch.org/global/2025/05/01/majorities-of-americans-support-several-but-not-all-types-of-foreign-aid/

[107] Eckenwiler L, Munoz-Beaulieu I, Perez R, Luneta M, Hyppolite SR, Schwartz L, et al. Thinking through abrupt closure in humanitarian assistance: Key ethical considerations in seemingly impossible conditions. PLOS Glob Public Health. 2025;5(6):e0004656. doi: 10.1371/journal.pgph.0004656 40465568 PMC12136286

[108] Stein F. Risky business: COVAX and the financialization of global vaccine equity. Global Health. 2021;17(1):112. doi: 10.1186/s12992-021-00763-8 34544439 PMC8451387

[109] Lexchin J. Profits First, Health Second: The Pharmaceutical Industry and the Global South Comment on “More Pain, More Gain! The Delivery of COVID-19 Vaccines and the Pharmaceutical Industry’s Role in Widening the Access Gap”. Int J Health Policy Manag. 2024;13:8471. doi: 10.34172/ijhpm.2024.8471 39099498 PMC11270595

[110] Pfeiffer J. The end of USAID reveals to folly of the NGO global health model. Science Politics; 2026 [07/02/2026]. Available from: https://sciencepolitics.org/2026/01/21/the-end-of-usaid-reveals-the-folly-of-the-ngo-global-health-model/

[111] Keynejad RC, Deraz O, Ingenhoff R, Eick F, Njie H, Graff S, et al. Decoloniality in global health research: ten tasks for early career researchers. BMJ Glob Health. 2023;8(11):e014298. doi: 10.1136/bmjgh-2023-014298 38007227 PMC10679997

[112] Saleh S, Campos Rivera PA, Lee K, Khor SK, Kyobutungi C. Global health reimagined: proposed functions, structures, and forms from five regions. Lancet Glob Health. 2025;13(11):e1801–2. doi: 10.1016/S2214-109X(25)00406-1 41015055

[113] Hatcher AM, Metheny N, Dunkle KL, Fielding-Miller R. A refusal to abandon HIV science. AIDS. 2025;39(6):768–70. doi: 10.1097/QAD.0000000000004188 40176537

[114] Kent L. These former USAID staff are working to match donors to urgent, lifesaving aid projects that had their funding slashed. 2025 [23/06/2025]. Available from: https://edition.cnn.com/2025/06/22/africa/former-usaid-staff-matching-donors-intl

[115] Horton R. Offline: Those one should not forgive. Lancet. 2025;406(10511):1454. doi: 10.1016/S0140-6736(25)02001-X 41046138

[116] Balabanova D, McKee M, Hutchinson E, Stoeva P, Spicer N. Announcing the Lancet Global Health Commission on anti-corruption in health: a call for a novel approach. Lancet Glob Health. 2025;13(8):e1341–2. doi: 10.1016/S2214-109X(25)00215-3 40712606

[117] Cavalcanti DM, de Oliveira Ferreira de Sales L, da Silva AF, Basterra EL, Pena D, Monti C, et al. Evaluating the impact of two decades of USAID interventions and projecting the effects of defunding on mortality up to 2030: a retrospective impact evaluation and forecasting analysis. Lancet. 2025;406(10500):283–94. doi: 10.1016/S0140-6736(25)01186-9 40609560 PMC12274115

[118] Mueller BNIH. Memo Pauses Cancellations of Medical Research Grants. 2025 [27/06/2025]. Available from: https://www.nytimes.com/2025/06/25/science/nih-grant-terminations-halted.html

[119] Wartiainen F, Boer B, Pauw P, Romijn H. Addressing the Twin Crises of Debt and Climate: Exploring Unconditional Debt Cancellation. Development. 2024;67(3–4):187–96. doi: 10.1057/s41301-025-00424-y

[120] The Pontifical Academy of Social Sciences. The Jubilee Report: A Blueprint for Tackling the Debt and Development Crises and Creating the Financial Foundations for a Sustainable People-Centered Global Economy. 2025 [26/10/2025]. Available from: https://www.pass.va/content/dam/casinapioiv/pass/pdf-booklet/2025_jubilee_report.pdf

[121] Rasanathan K, Cloete K, Gitahi G, Gómez-Dantés O, Saminarsih D, Swaminathan S, et al. Functions of the global health system in a new era. Nat Med. 2025;31(11):3605–8. doi: 10.1038/s41591-025-03936-9 40935857

[122] Krugman D, Smith J, Abimbola S. Global health after USAID cuts. Lancet. 2025;406(10520):2628. doi: 10.1016/S0140-6736(25)02018-5 41352966

[123] Kaunda Mwansa J, Hartmann M, Salazar M, Chibesakunda M, Vlahakis N, Ekström AM, et al. From shock to resilience in GBV and SRH services: the role of research. BMJ Glob Health. 2026;11(1):e021152. doi: 10.1136/bmjgh-2025-021152 41506753 PMC13059891

[124] WHO. Civil society shapes global health at WHA78. 2025 [23/06/2025]. Available from: https://www.who.int/news/item/19-06-2025-civil-society-shapes-global-health-at-wha78

[125] Dafallah A, Witter S, ReBUILD for Resilience Consortium. Diaspora as partners: strengthening resilience of health systems and communities amidst aid volatility. BMJ Glob Health. 2025;10(6):e019622. doi: 10.1136/bmjgh-2025-019622 40537273 PMC12182014

[126] Kaseya J, Keita D, Janabi MY, Khosla R. Africa’s future depends on investing in women, children, adolescents: Devex. 2026 [16/02/2026]. Available from: https://www.devex.com/news/africa-s-future-depends-on-investing-in-women-children-adolescents-111874

[127] Hessini L. Financing for gender equality and women’s rights: the role of feminist funds. Gend Dev. 2020;28(2):357–76. doi: 10.1080/13552074.2020.1766830

[128] Avakyan Y, Bywater K, Gleason A, Gordon C, Johansson S. Gender mainstreaming & other development fantasies. Gender and development: Perspectives from Australia and the Pacific. Springer; 2026. p. 303–27.

[129] Salles A, Banerjee AT, Caceres W, Mamas M, Blackstock O. Why and how academic medicine must champion diversity, equity, inclusion, and accessibility. Lancet. 2025;405(10494):2033–6. doi: 10.1016/S0140-6736(25)00575-6 40228510

[130] DefendResearch. Webinar Series. 2026 [15/02/2026]. Available from: https://www.defendresearch.org/events/webinar-series

[131] Schiff L, Meadows A, Mitchell C, Rouhi S, Suber P. Declaration To Defend Research Against U.S. Government Censorship, V.1.1. 2025 [15/02/2026]. Available from:https://docs.google.com/document/d/1cC6Vu_CAtgxSck8be_hmzSu-w2DsaJ02Nw5oeayAg/edit?tab=t.0

[132] Ahuja M. Exclusive: Iconiq Announces $100M Women’s Health Initiative With Melinda French Gates And Other Donors. 2025 [20/10/2025]. Available from: https://www.forbes.com/sites/maneetahuja/2025/09/29/iconiq-announces-100m-womens-health-initiative-alongside-melinda-french-gates-and-other-donors/

[133] Forbes M. Melinda French Gates Backs $100 Million Bet On Women’s Health Breakthroughs. 2025 [20/10/2025]. Available from: https://www.forbes.com/sites/moiraforbes/2025/09/10/melinda-french-gates-backs-100-million-bet-on-womens-health-breakthroughs/

[134] Cheng R. Reimagining women’s health is a global imperative. Br Med J Publish Group. 2025. 10.1136/bmj.r1537 40738619

